# Effect of Additional Motivational Interviewing on Smoking Abstinence for 1-Year among Korean Adolescents: Results from A Comparative Retrospective Study in Quitline

**DOI:** 10.3390/ijerph17218025

**Published:** 2020-10-31

**Authors:** Thi Phuong Thao Tran, Jinju Park, Eunjung Park, Sang Hwa Shin, Yu-Jin Paek, Yun Hee Kim, Min Kyung Lim

**Affiliations:** 1 Department of Cancer Control and Population Health, Graduate School of Cancer Science and Policy, National Cancer Center, Goyang 10408, Korea; 1906103@ncc.re.kr (T.P.T.T.); jinjupark@ncc.re.kr (J.P.); 2 Division of Cancer Prevention & Early Detection, National Cancer Control Institute, National Cancer Center, Goyang 10408, Korea; eunjungpark@ncc.re.kr (E.P.); quit@ncc.re.kr (S.H.S.); 3 Department of Family Medicine, Health Promotion Center, Hallym University Sacred Heart Hospital, Anyang 14068, Korea; samsumok@gmail.com; 4 Department of Nursing, Pukyong National University, Busan 48513, Korea; pumadrum@hanmail.net

**Keywords:** motivational enhancement, adolescent, smoking abstinence, Quitline, self-efficacy, Korea

## Abstract

The aim of this study was to evaluate the effect of additional motivational enhancement through telephone-based counseling on short- and long-term smoking abstinence among Korean adolescents. Methods: A comparative retrospective study was conducted based on the longitudinal follow up in Quitline from 2010 to 2017. A total of 533 and 178 adolescent smokers voluntarily participated in the 1-year quitting counseling only (group A, who were ready to quit) and the additional 4-week motivational interviewing before 1-year quitting counseling (group B, who were ambivalent about quitting), respectively. The outcomes were self-reported continuous abstinence at 30-day, 6-month, and 1-year follow up. Logistic regression was applied to estimate the effect of potential factors, including motivational enhancement, on cessation outcome. Results: At baseline, adolescents in group B had a lower motivation to quit than those in group A (*p* < 0.001). The successful quit rates at 30-day, 6-month, and 1-year follow up were 37.2%, 12.8%, and 11.4% in group A and 33.7%, 15.2%, and 11.2% in group B, respectively. After adjusting factors as appropriate, successful quit rates in group B were not significantly different from the rates in group A. Higher self-efficacy increased the successful quit rate at 30-day, 6-month, and 1-year follow up, similar in subgroup analysis by gender. Never-drinking showed significant association with 30-day successful quit in the whole population and among boys. The lower number of smoking triggers was associated with an increased 30-day successful quit rate among boys only. Conclusions: Counseling for motivational enhancement could be a promising approach for better quitting outcomes. Improving self-efficacy and eliminating smoking triggers should be continuously strengthened during the quitting process.

## 1. Introduction

The addictive nature of nicotine is usually underestimated among adolescents, and their quitting attempt is rarely planned and assisted, and their successful quit rate is lower than that in adult smokers [1,2,3,4,5]. Moreover, tobacco use among adolescents and their subsequent success in quitting could be influenced by a multitude of factors such as demographic factors, individual perception and behavior, social and political environments, and cultural backgrounds [6,7,8]. Therefore, the influential factors on successful quitting among adolescents should be investigated to suggest appropriate interventions accordingly [9].

Several previous studies, including meta-analyses, have shown that self-efficacy for quitting or self-identification as a smoker was suggested as a necessary precursor to raise intention to quit. Moreover, motivational enhancement plays a crucial role in successful cessation among adolescents based on social cognitive approaches, including cognitive behaviors, motivational enhancement, and social influences as the main elements [7,9,10,11,12]. Therefore, self-efficacy, which has been integrated as a central component of several cessation programs for adolescents, and motivational enhancement, identified as a promising strategy, has resulted in prolonged abstinence in several experimental studies [10,12].

However, there has been insufficient evidence demonstrating the effectiveness of each element of social cognitive approaches, including self-efficacy and motivational enhancement [6,10]. The nature of motivational enhancement varies across studies, and the effectiveness varies in providers, populations, and settings, including social contexts and policy influences [7,10,11,12]. Furthermore, motivational interviewing is a directive and client-centered counseling technique for eliciting self-motivational statements to positively change interviewees’ behaviors and serves as a crucial prelude to further therapeutic work for clients with ambivalence and low readiness [13]. It has been proven to be effective in a meta-analysis of 72 randomized controlled trials in significantly modifying several behaviors, such as weight control, reduced alcohol use, cholesterol level, and systolic control [14]. Even in people with physiological or psychological diseases, the effectiveness of motivational interviewing has the same effects [14,15,16]. For smoking cessation, compared to usual care or brief advice, motivational interviewing results in modestly successful quit rates [17,18]. In addition, programs including a motivational enhancement component have been proven to enhance quit rates for adolescents [6]. A randomized clinical trial among 162 adolescents suggested motivational interviewing could be an efficacious prelude to more intensive smoking intervention [18]. However, evidence on the effectiveness of these elements is insufficient in real-world settings. Hence, further studies are required in this field, specifically in Asia, where limited data are available, and the smoking prevalence of adolescents is still high [7,12].

In Korea, although the prevalence of tobacco use has decreased in recent years (with the reinforcement of tobacco control measures including price increases), its level is still significant (smoking prevalence of 3.8% and 2.1% in boys and girls in middle school students, and 14.1% and 5.1% in high school students, respectively) [19,20]. Furthermore, the prevalence might be higher than that reported from a national questionnaire survey regarding the gap between the biologically verified prevalence and the urine cotinine analysis [21]. Because adolescent tobacco use is usually considered to be a delinquent behavior to be corrected rather than an addictive behavior to be supported, some adolescent smokers could be reluctant to report their tobacco use [22]. The Nationwide Quitline in Korea (hereafter referred to as Quitline) was launched as a national cessation program in 2006 and suggested as an appropriate channel to support the quitting attempts of adolescents considering the anonymity and accessibility of the service. Since 2010, a specific quit protocol for adolescents has been applied. It adapts motivational interviewing as an important intervention strategy.

Hence, this study aimed to evaluate the effect of additional motivational interviewing through telephone-based counseling on short- and long-term smoking abstinence among Korean adolescents based on real-world Quitline settings. Additionally, the effect of baseline self-efficacy on successful quitting was investigated.

## 2. Methods

### 2.1. Quitline Protocol for Adolescent Smokers

In Korea, a specific cessation protocol targeting adolescents has been available in the service of Quitline since 2010, which offers highly accessible, confidential, and intensive service to help adolescents who smoke quit tobacco use through telephone counseling by trained quitting coaches.

Quitline has two different arms of counseling depending on the motivation of smokers to quit. One arm offers 1-year counseling for quitting and maintenance for smokers who are ready to quit (group A). Here, at least 21 calls are given during 1 year—14 calls for intensive counseling to overcome withdrawal symptoms during the first 4 weeks and 7 calls for quitting maintenance counseling and modification of other health behaviors during the remaining 11 months. Another arm offers an additional 4 weeks of counseling for motivational interviewing before initiating 1-year counseling for quitting and maintenance (group B), which is applicable for adolescent smokers who have ambivalent or insecure feelings in their motivation to quit. Before involvement in the Quitline program, quitting coaches evaluate adolescents’ self-efficacy using Shin’s instrument [23], and assess their readiness to quit by asking the question “Are you ready to quit?”. If adolescents are ready to quit immediately, quit coaches allow them to join group A. In cases in which adolescents are ambivalent about quitting, they are encouraged to be involved in the 4-week motivational interviewing prior to the 1-year quitting program (group B). Adolescents choose one of two groups depending on their own decision and considering the quitting coaches’ recommendation. During the 4-week motivational enhancement, 8 calls (2 calls per week) are made to the smokers to build rapport and ask for personal reasons to quit in the 1st week; assessing nicotine addiction level and correcting misconceptions about tobacco use in the 2nd week; understanding the harm of tobacco use and benefits of quitting in the 3rd week; and indicating the obstacles to quitting and how to address these in the 4th week. Thereafter, 1-year counseling for quitting and maintenance is provided for those who are willing to set a quit date after completing a 4-week motivational enhancement. Nicotine replacement therapy and prescribed drugs are not included in these protocols.

The quitting coaches in the adolescent-specialized quitting protocol are persons with at least 2 years of experience working in Quitline counseling on smoking cessation. They must complete a comprehensive 2-month training course focusing on tobacco-related issues among adolescents, including: (1) understanding the specific characteristics of adolescent smokers and their smoking patterns; (2) short-term benefits of quitting for adolescents; (3) harm due to smoking initiation and ongoing smoking on adolescents’ health; (4) reasons/triggers of smoking and effective preventive measures; (5) communication skills with adolescents. The training course also addresses effective ways of motivational interviewing, including: (1) understanding ambivalence and expressing empathy; (2) facilitating exploration of stage-specific motivational conflict; (3) helping direct confrontation; (4) promoting self-efficacy; and (5) affirming the decision to quit. Two to four weeks practice with roll play interviews is undertaken after completion of the training course. Furthermore, the capacity of quitting coaches’ is also evaluated weekly through recorded counseling conversations by the evaluation committee, which is composed of experts.

### 2.2. Study Design and Population

**Figure 1:**
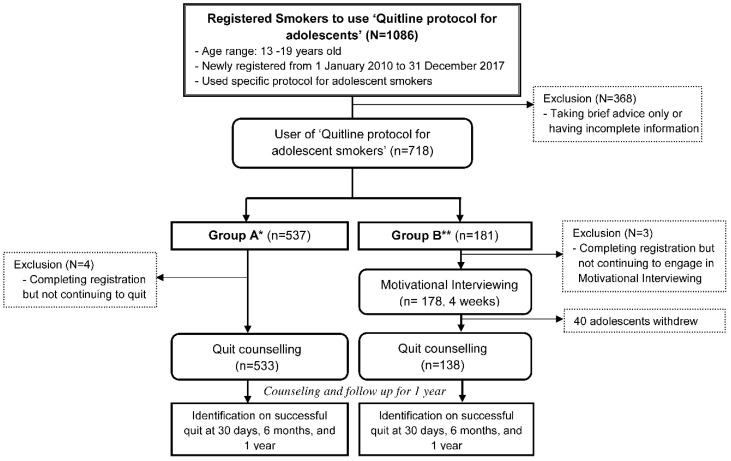
Service protocol for counseling, process for follow up, and subjects included.

A retrospective comparative study was conducted based on the longitudinal follow up among adolescent smokers who voluntarily called and registered in Quitline for counseling to quit. As shown in Figure 1, 1086 adolescent smokers aged between 13 and 19 years, who newly registered in “Quitline protocol for adolescent smokers” from 1 January 2010, to 31 December 2017, were enrolled. After excluding 368 adolescent smokers who decided to receive brief advice only or had incomplete baseline information, 537 and 181 adolescent smokers voluntarily decided to participate in the 1-year quitting and maintenance counseling only (group A) and the additional 4-week motivational enhancement before 1-year quitting and maintenance counseling (group B), respectively. Adolescents self-selected the group based on their willingness and recommendations from quitting coaches after assessing their readiness to quit. Four and three adolescents in group A and group B who did not want to continue the cessation program were excluded, respectively. Forty adolescents of the 178 in group B withdrew their participation during the 4-week motivational enhancement. Finally, 533 and 138 adolescent smokers in group A and group B correspondingly decided to quit smoking and engaged in the 1-year quitting and maintenance protocol.

### 2.3. Measures

The baseline information on gender (boys/girls), age (13–16/17–19 years), age at smoking initiation (13 or less/14–16/17–19 years), daily cigarette consumption (the number of cigarette per day, less than 10/10–19/20 and more), nicotine dependence (0–3/4–6/7–10) [24], alcohol consumption (never/ever), quitting supporters (none/peers/adults), specific triggers for smoking, and the reasons provided to quit smoking were obtained. For multivariate analysis, age at smoking initiation (13 or less/14–19 years) and nicotine dependence (0–3/4–10) were categorized into two groups.

Self-efficacy for quitting was measured by an instrument including eight statements [23]. Participants would answer “Yes” or “No” for each statement and the maximum score gained for each statement was 1 point per answer. The sum of the scores of self-efficacy was calculated and categorized into 3 groups (0–2/3–5/6–8). Triggers for smoking included drinking coffee or alcohol; seeing others smoking in real life or on television; being stressed, excited, or tired; being alone; and other daily activities such as after waking, before going to bed, after taking a shower, during phone calls, and while playing a game. The number of triggers reported was categorized (1/2/3 or more). The reasons for quitting smoking, including identifying themselves as smokers, having the self-confidence to quit, personal health issues related to tobacco use, economic burden from buying tobacco, having a good social relationship, getting positive public attention, and recommendation to quit from their surroundings, were investigated, counted, and categorized (0–2/3–4/5 or more).

Additionally, 30-day, 6-month, and 1-year continuous abstinence rates were assessed through telephone calls after initiating the 1-year quit protocol based on self-reported data. Adolescent smokers who withdrew their participation during the 4-week motivational interviewing, smoked even once puff of smoking, or were lost to follow up (no response to 3 phone calls) were considered to be failed participants.

### 2.4. Statistical Analysis

A chi-squared test was applied to identify the difference between the frequency distribution of each variable and the cessation groups. Logistic regression was applied to calculate the odds ratios (ORs) and confidence intervals (CIs) of the cessation groups and other potential factors on cessation outcomes at 30-day, 6-month, and 1-year follow up. Analyses were performed for girls and boys, separately. All statistical analyses were performed using STATA (version 14.0) software (Stata Corp, College Station, Texas, USA), and *p* < 0.05 was considered statistically significant.

### 2.5. Ethics

All study protocols and processes were approved by the Institutional Review Board of the National Cancer Center of Korea (NCC 2017-0143). Verbal consent by telephone was obtained from all participants with the waiver of written informed consent.

## 3. Results

The majority of adolescent smokers were boys, aged 17–19 years, initiated smoking after the age of 14 years, smoked less than 20 cigarettes per day, had low nicotine dependence, and had adult supporters. The frequency distribution of all variables did not show any significant difference between the two cessation groups, except that the number of smoking triggers and the number of reasons for quit were higher in group A than in group B (Table 1).

The successful quit rates at 30-day, 6-month, and 1-year follow up were 37.2%, 12.8%, and 11.4% in group A, and 33.7%, 15.2%, and 11.2% in group B, respectively. After adjusting for other covariates, the successful quit rates were not significantly different between the cessation groups (group A, the 1-year quitting and maintenance counseling only; group B, the additional 4-week motivational interviewing before 1-year quitting and maintenance counseling). Higher self-efficacy significantly increased the successful quit rate at 30-day, 6-month, and 1-year follow up, while never-drinking increased the rate at 30-day follow up only in multivariate models (Table 2).

Having a lower number of cigarettes smoked daily, lower nicotine dependence, no experience in drinking, lower number of smoking triggers, and adult quitting supporter were associated with successful quitting in age- and gender-adjusted models; however, their effects were not observed in multivariate models (Table 2).

Similar results were observed in the subgroup analysis by gender, although the lower number of smoking triggers was an additional factor significantly associated with increased successful quit rates at 30-day follow up among boys only (Table 3 and Table 4).

## 4. Discussion

As suggested in the previous evidence, motivational enhancement has been identified as an efficacious approach for adolescent smokers to produce a better outcome in their quitting attempt [6,7]. Furthermore, Quitline has been recommended as an effective service to help smokers quit due to it accessibility, convenience, and confidentiality [25]. Therefore, based on these theoretical and experimental studies, an additional program of 4-week motivational interviewing before the 1-year quitting protocol was developed and applied for adolescent smokers who were ambivalent about quitting and contacting Quitline in Korea. The results suggest that adolescent smokers who were ambivalent about quitting at baseline who underwent 4-week motivational interviewing have short-and long-term successful quit rates that were not significantly different from the rates of those having relatively higher motivation and starting 1-year quitting protocol without motivational interviewing. This indicates that the successful quit rate could be increased through the additional motivational interviewing for adolescents with less motivation for quitting. Although there are no studies comparing the successful quit rates among those having different motivation levels at baseline as in our study, a randomized factorial experiment revealed that motivation intervention can enhance quitting attempts for smokers initially unwilling to quit [26]. A previous meta-analysis study and several systematic reviews also indicated that motivation interviewing could significantly improve a successful quit rate for adolescents [6,7,12,27]. Thus, additional motivational interviewing in real-world Quitline settings could be an effective and promising approach to improve the cessation outcomes among adolescent smokers with or without motivation to quit smoking, although further studies with a larger population are needed.

Our study reinforces the fact that self-efficacy was a determinant of both short- and long-term successful quitting among Korean adolescents. This result replicates a previous study in European adolescents [28] and a 3-year longitudinal study of high school students in Taiwan [29], which revealed that self-efficacy was a strong predictor of smoking abstinence. Moreover, another theoretical study concluded that self-efficacy was an important indicator to provide an appropriate approach to smoking cessation counseling for adolescent smokers [30]. Therefore, improving self-efficacy for adolescents during behavioral counseling plays a critical role in the quitting process, and should be continuously strengthened during long-term smoking cessation.

In this study, the significant association between never-drinking and 30-day successful quitting was observed. Similarly, the negative association between alcohol consumption and smoking abstinence among adolescents has been well established in a series of cross-sectional surveys [28,31] and longitudinal studies [28,29], and a review [32]. A review of human laboratory studies [33] and population-based studies [34,35] demonstrated that alcohol drinking could increase the urge for smoking because it is a delinquent behavior that usually coexists with smoking and synergistically increases the addiction to tobacco use.

Our study also investigated other smoking triggers among adolescents such as after waking, emotions (e.g., happy, stressed, angry), and contextual temptation (e.g., seeing someone smoking), as suggested by a previous study [35]. The major smoking triggers in our study consisted of “after meal,” “after waking,” “seeing someone smoking,” and “being tired, stressed, or excited.” Our results revealed that having a higher number of smoking triggers could significantly decrease the successful quit rate at 30-day follow up only among boys. It has been demonstrated that the appearance of the nicotine receptor, correlated with a craving to smoke, reached the highest level in the first 4 weeks of abstinence and subsequently decreased [36]. This is a novel finding from the present study, which has not been shown in previous studies, regarding “stress” and “being with smokers” as triggers for smoking [31,37,38]. Thus, our study results suggest that understanding these smoking triggers, such as drinking, “after meal”, “after waking,” and “seeing someone smoking”, might be considered an essential component of quitting counseling to minimize relapse during short-term smoking abstinence. Furthermore, there was no clear explanation nor previous studies that indicate that highly motivated adolescent smokers have a higher number of triggers. However, one of the possible explanations is that adolescents who were motivated were more likely to recognize their triggers for smoking as one of the processes for their readiness to quit. This could also be supported by the result that the proportion of adolescent smokers reporting “after waking” as the trigger for smoking, which represents a level of addiction, was higher in group B than in group A.

Nicotine dependence was not associated with successful quitting in this study after controlling for other baseline factors. In contrast, heavy nicotine dependence (e.g., the number of cigarettes smoked daily) is one of the major obstacles in smoking cessation among adolescents, as suggested by prior longitudinal studies in European countries [28,37], and in the US [39,40], in which ORs ranged from 0.1 to 0.87, *p* < 0.05. In Asia, a significantly limited number of studies on the association between nicotine dependence and smoking cessation among adolescents have been conducted. A study conducted in Taiwan did not consider the effect of nicotine addiction on successful quitting [29]. Lim et al. showed the insignificant association between nicotine dependence and smoking relapse (HR = 1.13; 96% CI = 0.88–1.46) [22].

Our study suggests that social support might have a supportive impact on short-term smoking cessation for adolescents, although the impact was significant in the partially adjusted model. This result is consistent with the previous evidence, in which social support has a critical role in increasing successful tobacco cessation and maintaining smoking abstinence [12,41]. Thus, strengthening the motivation to quit by emotional and physical support from family members and society could be considered during the smoking abstinence process for adolescent smokers who have less motivation to quit.

The current research was the first retrospective, comparative study that applied motivational interviewing in a real-world Quitline setting and assessed its effectiveness. We also identified other potential factors associated with smoking cessation among adolescent smokers, which provided more valuable evidence in the context of the significant knowledge gap in this area in Asia. However, this study has several limitations. First, the smoking outcome was based on self-reported data without any confirmation by biological measures, although deceitful answers are likely to be rare during quitting attempts via telephone counseling, which can guarantee confidentiality without a face-to-face interview. Second, the follow up period for a successful quitting attempt was limited to 1 year, although it is a relatively longer period compared with previous studies in experimental settings [26,42,43]. Third, the current investigations were based on the first quit attempt of adolescent smokers who registered in Quitline. Subsequent quit attempts, which usually increase the successful quit rate, were not considered. Fourth, adolescent smokers in each cessation group could have a different level of motivation to quit. Smokers were not randomly assigned, but voluntarily selected their group based on the recommendations from quitting coaches after assessing their readiness to quit and on their willingness by self-estimated motivation. Furthermore, an objective measure of the level of skillfulness in motivational interviewing was not conducted, although evaluation of the coaches’ capacity and quality of counseling was performed on a weekly basis. However, this might not have interfered with the current findings because we did not see different results in the subgroup analysis by self-efficacy level and the number reasons to quit. Finally, school and family environments are among the most critical factors that strongly influence adolescents [8,44,45,46,47]. However, our study did not include these factors, including parental smokers, parental disapproval of smoking, and number of peer smokers. However, we assessed the impact of social supporters (e.g., family members, teachers, peers, and doctors) on successful quitting.

## 5. Conclusions

The current study suggested that additional counseling for motivational interviewing in the Quitline could contribute to better successful quitting attempts among adolescent smokers. Therefore, it might be a promising approach that can be applied in adolescent-focused intervention in real-world cessation programs. Additionally, improving self-efficacy and eliminating smoking triggers (e.g., alcohol drinking) plays a critical role that should be continuously strengthened during the smoking cessation process.

## Figures and Tables

* Group A includes 1-year counseling for quitting and maintenance. ** Group B includes 4-week counseling for motivational interviewing before 1-year counseling for quitting and maintenance.

**Table 1:** Baseline characteristics of adolescent Quitline users by cessation groups.

Baseline Characteristics	Total *n* (%)	Group A ^a^ *n* (%)	Group B ^b^ *n* (%)	*p*-Value ^c^
	711 (100.0)	533 (75.0)	178 (25.0)	
Gender				0.70
Boys	572 (80.5)	427 (80.1)	145 (81.5)	
Girls	139 (19.6)	106 (19.9)	33 (18.5)	
Age group				0.10
13–16	210 (29.5)	166 (31.1)	44 (24.7)	
17–19	501 (70.5)	367 (68.9)	134 (75.3)	
Age at smoking initiation				0.39
≤13	170 (24.1)	131 (24.6)	39 (22.5)	
14–16	482 (68.4)	365 (68.6)	117 (67.6)	
17–19	53 (7.5)	36 (6.8)	17 (9.8)	
Daily cigarette consumption				0.47
<10	276 (39.7)	212 (40.2)	64 (38.1)	
10–19	257 (37.0)	198 (37.6)	59 (35.1)	
≥20	162 (23.3)	117 (22.2)	45 (26.8)	
Nicotine dependence ^d^				0.62
0–3	440 (61.9)	335 (62.9)	105 (59.0)	
4–6	219 (30.8)	161 (30.2)	58 (32.6)	
7–10	52 (7.3)	37 (6.9)	15 (8.4)	
Alcohol consumption				0.46
Never	274 (39.0)	211 (39.8)	63 (36.6)	
Ever	428 (61.0)	319 (60.2)	109 (63.4)	
Supporter				0.82
None	152 (21.9)	114 (21.8)	38 (22.2)	
Peers	150 (21.6)	116 (22.1)	34 (19.9)	
Adults	393 (56.6)	294 (56.1)	99 (57.9)	
Number of triggers for smoking ^e^				0.005
1	207 (30.0)	138 (26.7)	69 (39.4)	
2	252 (36.5)	193 (37.4)	59 (33.7)	
≥3	232 (33.6)	185 (35.9)	47 (26.9)	
Self-efficacy ^f^				0.06
0–2	159 (22.4)	109 (20.5)	50 (28.1)	
3–5	314 (44.2)	236 (44.3)	78 (43.8)	
6–8	238 (33.5)	188 (35.3)	50 (28.1)	
Number of reasons to quit ^g^				<0.001
0–2	248 (34.9)	158 (29.6)	90 (50.6)	
3–4	271 (38.1)	217 (40.7)	54 (30.3)	
≥5	192 (27.0)	158 (29.6)	34 (19.1)	

^a.^ Group A offers 1-year counseling for quit and maintenance. ^b.^ Group B offers 4-week counseling for motivational interviewing and then 1-year counseling for quit and maintenance. ^c.^ Chi-squared test. ^d.^ Nicotine dependence: mild (0–3), moderate (4–6), and severe (7–10), using Fagerström Test for Nicotine Dependence. ^e.^ Triggers for smoking: drinking coffee or alcohol, seeing others smoking in real life or on television, being stressed, excited or tired, being alone, and other daily activities such as after walking, before going to bed, after taking a shower, during phone calls, and while playing a game. ^f.^ Self-efficacy: using Self-efficacy Scale. ^g.^ Reasons to quit: identifying themselves as smokers, having self-confidence for quitting, personal health issue related to tobacco use, economic burden from buying tobacco, having a good social relationship, getting positive public attention, recommendation to quit from their surroundings, and others.

**Table 2:** Odds ratios and confidence intervals of potential factors associated with successful quit among adolescent Quitline users (Overall).

Baseline Characteristics	Total	Successful Quit at 30 Days			Successful Quit at 6 Months			Successful Quit at 1 Year		
		*n* (%)	Model 1 ^a^	Model 2 ^b^	*n* (%)	Model 1 ^a^	Model 2 ^b^	*n* (%)	Model 1 ^a^	Model 2 ^b^
	*n* = 711	258 (36.3)	OR (95%CI)	OR (95%CI)	95 (13.4)	OR (95%CI)	OR (95%CI)	81 (11.4)	OR (95%CI)	OR (95%CI)
Gender										
Boys	572	210 (36.7)	1	1	76 (13.3)	1	1	63 (11.0)	1	1
Girls	139	48 (34.5)	0.912 (0.618–1.346)	0.937 (0.613–1.432)	19 (13.7)	1.029 (0.599–1.768)	1.119 (0.631–1.985)	18 (13.0)	1.198 (0.684–2.098)	1.337 (0.736–2.428)
Age group										
13–16	210	82 (39.1)	1	1	24 (11.4)	1	1	21 (10.0)	1	1
17–19	501	176 (35.1)	0.846 (0.607–1.180)	1.101 (0.760–1.596)	71 (14.2)	1.279 (0.781–2.096)	1.403 (0.830–2.373)	60 (12.0)	1.221 (0.722–2.066)	1.330 (0.758–2.332)
Age at smoking initiation										
≤13	170	63 (37.1)	1		20 (11.8)	1		17 (10.0)	1	
14–19	535	195 (36.5)	1.018 (0.704–1.470)		75 (14.0)	1.159 (0.676–1.990)		64 (12.0)	1.166 (0.653–2.082)	
Daily cigarette consumption										
<10	276	117 (42.4)	1		45 (16.3)	1		38 (13.8)	1	
10–19	257	81 (31.5)	0.631 (0.441–0.903)		30 (11.7)	0.654 (0.396–1.080)		26 (10.1)	0.686 (0.401–1.172)	
≥20	162	53 (32.7)	0.658 (0.438–0.989)		19 (11.7)	0.677 (0.381–1.206)		16 (9.9)	0.687 (0.370–1.278)	
Nicotine dependence ^c^										
0–3	440	182 (41.4)	1	1	67 (15.2)	1	1	58 (13.2)	1	1
4–10	271	76 (28.0)	0.557 (0.402–0.773)	0.903 (0.626–1.301)	28 (10.3)	0.625 (0.390–1.003)	0.989 (0.592–1.652)	23 (8.5)	0.599 (0.359–0.998)	0.994 (0.570–1.736)
Alcohol consumption										
Never	274	128 (46.7)	1	1	47 (17.2)	1	1	40 (14.6)	1	1
Ever	428	127 (29.7)	0.485 (0.353–0.668)	0.632 (0.445–0.897)	46 (10.8)	0.557 (0.357–0.868)	0.688 (0.425–1.112)	39 (9.1)	0.567 (0.352–0.912)	0.730 (0.434–1.226)
Supporter										
None	152	46 (30.3)	1		16 (10.5)	1		13 (8.6)	1	
Peers	150	51 (34.0)	1.189 (0.732–1.930)		22 (14.7)	1.470 (0.738–2.928)		18 (12.0)	1.450 (0.682–3.080)	
Adults	393	156 (39.7)	1.525 (1.020–2.279)		56 (14.3)	1.414 (0.783–2.555)		49 (12.5)	1.508 (0.792–2.872)	
Number of triggers for smoking ^d^										
1	207	93 (44.9)	1	1	36 (17.4)	1	1	31 (15.0)	1	1
2	252	89 (35.3)	0.671 (0.460–0.978)	0.801 (0.533–1.202)	34 (13.5)	0.741 (0.445–1.235)	0.960 (0.561–1.643)	30 (11.9)	0.761 (0.443–1.307)	1.006 (0.568–1.783)
≥3	232	64 (27.6)	0.471 (0.316–0.703)	0.667 (0.431–1.033)	21 (9.1)	0.461 (0.259–0.822)	0.696 (0.376–1.289)	16 (6.9)	0.407 (0.215–0.772)	0.607 (0.308–1.195)
Self-efficacy ^e^										
0–2	159	24 (15.1)	1	1	7 (4.4)	1	1	7 (4.4)	1	1
3–5	314	99 (31.5)	2.580 (1.570–4.238)	2.359 (1.420–3.920)	35 (11.2)	2.836 (1.228–6.551)	2.677 (1.145–6.259)	25 (8.0)	1.957 (0.826–4.640)	1.766 (0.734–4.246)
6–8	238	135 (56.7)	7.334 (4.419–12.170)	5.377 (3.155–9.166)	53 (22.3)	6.571 (2.892–14.931)	5.389 (2.289–12.690)	49 (20.6)	5.965 (2.614–13.610)	4.643 (1.955–11.030)
Number of reasons to quit ^f^										
0–2	248	87 (35.1)	1		34 (13.7)	1		30 (12.1)	1	
3–4	271	101 (37.3)	1.096 (0.765–1.570)		33 (12.2)	0.874 (0.523–1.461)		28 (10.3)	0.842 (0.487–1.456)	
≥5	192	70 (36.5)	1.062 (0.716–1.575)		28 (14.6)	1.069 (0.622–1.836)		23 (12.0)	0.993 (0.555–1.774)	
Cessation groups ^g^										
Group A	533	198 (37.2)	1	1	68 (12.8)	1	1	61 (11.4)	1	1
Group B	178	60 (33.7)	0.868 (0.607–1.242)	0.901 (0.606–1.340)	27 (15.2)	1.206 (0.744–1.956)	1.433 (0.854–2.402)	20 (11.2)	0.970 (0.567–1.661)	1.127 (0.634–2.003)

^a.^ Model 1: Adjusted for gender and age. ^b.^ Model 2: Adjusted for gender, age, nicotine dependence, alcohol consumption, number of triggers for smoking, self-efficacy, and cessation group. ^c.^ Nicotine dependence: mild (0–3), moderate (4–6), and severe (7–10), using Fagerström test for nicotine dependence. ^d.^ Triggers for smoking: drinking coffee or alcohol, seeing others smoking in real life or on television, being stressed, excited or tired, being alone, and other daily activities such as after walking, before going to bed, after taking a shower, during phone calls, and while playing a game. ^e.^ Self-efficacy: using Self-efficacy Scale. ^f.^ Reasons to quit: identifying themselves as smokers, having self-confidence for quitting, personal health issue related to tobacco use, economic burden from buying tobacco, having a good social relationship, getting positive public attention, recommendation to quit from their surroundings, and others. ^g.^ Cessation groups: Group A offers 1- year counseling for quit and maintenance; Group B offers 4 weeks counseling for motivational interviewing and then 1-year counseling for quitting and maintenance.

**Table 3:** Odds ratios and confidence intervals of potential factors associated with successful quit among adolescent Quitline users (Boys).

Baseline Characteristics	Boys	Successful Quit at 30 Days			Successful Quit at 6 Months			Successful Quit at 1 Year		
		*n* (%)	Model 1 ^a^	Model 2 ^b^	*n* (%)	Model 1 ^a^	Model 2 ^b^	*n* (%)	Model 1 ^a^	Model 2 ^b^
	*n* = 572	210 (36.7)	OR (95%CI)	OR (95%CI)	76 (13.3)	OR (95%CI)	OR (95%CI)	63 (11.0)	OR (95%CI)	OR (95%CI)
Age group										
13–16	171	69 (40.4)	1	1	20 (11.7)	1	1	17 (9.9)	1	1
17–19	401	141 (35.2)	0.802 (0.555–1.158)	1.112 (0.732–1.687)	56 (14.0)	1.226 (0.710–2.114)	1.298 (0.722–2.336)	46 (11.5)	1.174 (0.652–2.112)	1.23 (0.652–2.321)
Age at smoking initiation										
≤13	141	52 (36.9)	1		16 (11.4)	1		14 (9.9)	1	
14–19	425	158 (37.2)	1.063 (0.710–1.591)		60 (14.1)	1.239 (0.681–2.254)		49 (11.5)	1.147 (0.605–2.174)	
Daily cigarette consumption										
<10	217	92 (42.4)	1		36 (16.6)	1		29 (13.4)	1	
10–19	206	67 (32.5)	0.668 (0.448–0.997)		22 (10.7)	0.581 (0.327–1.032)		18 (8.7)	0.604 (0.323–1.131)	
≥20	134	45 (33.6)	0.691 (0.441–1.082)		17 (12.7)	0.723 (0.388–1.349)		15 (11.2)	0.811 (0.417–1.577)	
Nicotine dependence ^c^										
0–3	352	145 (41.2)	1	1	52 (14.8)	1	1	43 (12.2)	1	1
4–10	220	65 (29.6)	0.608 (0.424–0.871)	1.032 (0.685–1.554)	24 (10.9)	0.692 (0.412–1.162)	1.085 (0.615–1.914)	20 (9.1)	0.707 (0.403–1.240)	1.182 (0.637–2.192)
Alcohol consumption										
Never	219	104 (47.5)	1	1	36 (16.4)	1	1	29 (13.2)	1	1
Ever	345	104 (30.1)	0.485 (0.339–0.694)	0.630 (0.425–0.933)	38 (11.0)	0.599 (0.363–0.989)	0.707 (0.413–1.211)	32 (9.3)	0.646 (0.375–1.113)	0.788 (0.438–1.417)
Supporter										
None	130	42 (32.3)	1		13 (10.0)	1		10 (7.7)	1	
Peers	119	41 (34.5)	1.088 (0.642–1.846)		16 (13.5)	1.410 (0.647–3.072)		12 (10.1)	1.354 (0.562–3.261)	
Adults	311	123 (39.6)	1.368 (0.887–2.109)		46 (14.8)	1.566 (0.815–3.009)		40 (12.9)	1.774 (0.859–3.665)	
Number of triggers for smoking ^d^										
1	174	78 (44.8)	1	1	30 (17.2)	1	1	25 (14.4)	1	1
2	200	77 (38.5)	0.770 (0.510–1.164)	0.841 (0.540–1.311)	26 (13.0)	0.718 (0.406–1.269)	0.856 (0.472–1.551)	22 (11.0)	0.737 (0.399–1.361)	0.871 (0.459–1.652)
≥3	180	45 (25.0)	0.414 (0.263–0.651)	0.558 (0.340–0.917)	17 (9.4)	0.487 (0.257–0.924)	0.753 (0.379–1.496)	13 (7.2)	0.454 (0.223–0.924)	0.673 (0.315–1.436)
Self-efficacy ^e^										
0-2	123	17 (13.8)	1	1	5 (4.1)	1	1	5 (4.1)	1	1
3–5	253	81 (32.0)	2.916 (1.637–5.194)	2.649 (1.465–4.789)	29 (11.5)	3.151 (1.187–8.367)	3.053 (1.131–8.239)	20 (7.9)	2.080 (0.760–5.690)	1.939 (0.696–5.403)
6–8	196	112 (57.1)	8.232 (4.577–14.808)	6.006 (3.228–11.174)	42 (21.4)	6.746 (2.58–17.644)	5.894 (2.155–16.117)	38 (19.4)	5.912 (2.249–15.539)	5.001 (1.807–13.835)
Number of reasons to quit ^f^										
0–2	193	70 (36.3)	1		28 (14.5)	1		24 (12.4)	1	
3–4	219	82 (37.4)	1.051 (0.703–1.570)		26 (11.9)	0.795 (0.448–1.409)		22 (10.1)	0.787 (0.426–1.454)	
≥5	160	58 (36.3)	1.002 (0.648–1.550)		22 (13.8)	0.937 (0.513–1.712)		17 (10.6)	0.835 (0.432–1.617)	
Cessation groups ^g^										
Group A	427	162 (37.9)	1	1	52 (12.2)	1	1	46 (10.8)	1	1
Group B	145	48 (33.1)	0.825 (0.554–1.230)	0.845 (0.542–1.317)	24 (16.6)	1.408 (0.831–2.386)	1.637 (0.928–2.888)	17 (11.7)	1.085 (0.599–1.964)	1.211 (0.639–2.294)

^a.^ Model 1: Adjusted for age. ^b.^ Model 2: Adjusted for age, nicotine dependence, alcohol consumption, number of triggers for smoking, self-efficacy, and cessation group. ^c.^ Nicotine dependence: mild (0–3), moderate (4–6), and severe (7–10), using Fagerström test for nicotine dependence. ^d.^ Triggers for smoking: drinking coffee or alcohol, seeing others smoking in real life or on television, being stressed, excited or tired, being alone, and other daily activities such as after walking, before going to bed, after taking a shower, during phone calls, and while playing a game. ^e.^ Self-efficacy: using Self-efficacy Scale. ^f.^ Reasons to quit: identifying themselves as smokers, having self-confidence for quitting, personal health issue related to tobacco use, economic burden from buying tobacco, having a good social relationship, getting positive public attention, recommendation to quit from their surroundings, and others. ^g^^.^ Cessation groups: Group A offers 1-year counseling for quit and maintenance; Group B offers 4 weeks counseling for motivational interviewing and then 1-year counseling for quit and maintenance.

**Table 4:** Odds ratios and confidence intervals of potential factors associated with successful quit among adolescent Quitline users (Girls).

Baseline characteristics	Girls	Successful Quit at 30 Days			Successful Quit at 6 Months			Successful Quit at 1 Year		
		n (%)	Model 1 ^a^	Model 2 ^b^	n (%)	Model 1 ^a^	Model 2 ^b^	n (%)	Model 1 ^a^	Model 2 ^b^
	n = 139	48 (34.5)	OR (95%CI)	OR (95%CI)	19 (13.7)	OR (95%CI)	OR (95%CI)	18 (12.95)	OR (95%CI)	OR (95%CI)
Age group										
13–16	39	13 (33.3)	1	1	4 (10.3)	1	1	4 (10.3)	1	1
17–19	100	35 (35.0)	1.077 (0.493–2.355)	1.233 (0.518–2.934)	15 (15.0)	1.544 (0.479–4.98)	1.613 (0.462–5.627)	14 (14.0)	1.424 (0.438–4.629)	1.532 (0.422–5.561)
Age at smoking initiation										
≤13	29	11 (37.9)	1		4 (13.8)	1		3 (10.3)	1	
14–19	110	37 (33.6)	0.782 (0.315–1.938)		15 (13.6)	0.82 (0.230–2.921)		15 (13.6)	1.227 (0.306–4.925)	
Daily cigarette consumption										
<10	59	25 (42.4)	1		9 (15.3)	1		9 (15.3)	1	
10–19	51	14 (27.5)	0.497 (0.220–1.121)		8 (15.7)	0.985 (0.345–2.81)		8 (15.7)	0.999 (0.350–2.853)	
≥20	28	8 (28.6)	0.548 (0.207–1.445)		2 (7.1)	0.432 (0.087–2.149)		1 (3.6)	0.207 (0.025–1.724)	
Nicotine dependence ^c^										
0–3	88	37 (42.1)	1	1	15 (17.1)	1	1	15 (17.1)	1	1
4–10	51	11 (21.6)	0.371 (0.168–0.823)	0.570 (0.240–1.354)	4 (7.8)	0.392 (0.122–1.264)	0.548 (0.152–1.979)	3 (5.9)	0.290 (0.079–1.061)	0.395 (0.095–1.636)
Alcohol consumption										
Never	55	24 (43.6)	1	1	11 (20.0)	1	1	11 (20.0)	1	1
Ever	83	23 (27.7)	0.495 (0.242–1.015)	0.691 (0.303–1.577)	8 (9.6)	0.428 (0.160–1.147)	0.524 (0.162–1.694)	7 (8.4)	0.369 (0.133–1.024)	0.439 (0.125–1.537)
Supporter										
None	22	4 (18.2)	1		3 (13.6)	1		3 (13.6)	1	
Peers	31	10 (32.3)	2.139 (0.571–8.010)		6 (19.4)	1.514 (0.334–6.866)		6 (19.4)	1.515 (0.334–6.862)	
Adults	82	33 (40.2)	3.050 (0.946–9.840)		10 (12.2)	0.886 (0.221–3.552)		9 (11.0)	0.785 (0.193–3.193)	
Number of triggers for smoking ^d^										
1	33	15 (45.5)	1	1	6 (18.2)	1	1	6 (18.2)	1	1
2	52	12 (23.1)	0.361 (0.141–0.927)	0.656 (0.219–1.964)	8 (15.4)	0.829 (0.259–2.656)	2.454 (0.562–10.71)	8 (15.4)	0.826 (0.258–2.645)	3.248 (0.693–15.217)
≥3	52	19 (36.5)	0.694 (0.285–1.688)	1.180 (0.433–3.218)	4 (7.7)	0.380 (0.098–1.467)	0.705 (0.164–3.038)	3 (5.8)	0.278 (0.064–1.202)	0.560 (0.116–2.706)
Self-efficacy ^e^										
0–2	36	7 (19.4)	1	1	2 (5.6)	1	1	2 (5.6)	1	1
3–5	61	18 (29.5)	1.768 (0.652–4.798)	1.626 (0.576–4.592)	6 (9.8)	1.976 (0.374–10.429)	1.828 (0.339–9.860)	5 (8.2)	1.599 (0.292–8.762)	1.457 (0.255–8.331)
6–8	42	23 (54.8)	5.088 (1.818–14.238)	3.584 (1.182–10.866)	11 (26.2)	6.347 (1.294–31.143)	5.713 (1.040–31.381)	11 (26.2)	6.286 (1.282–30.82)	6.035 (1.059–34.402)
Number of reasons to quit ^f^										
0–2	55	17 (30.9)	1		6 (10.9)	1		6 (10.9)	1	
3–4	52	19 (36.5)	1.287 (0.576–2.874)		7 (13.5)	1.271 (0.396–4.073)		6 (11.5)	1.065 (0.320–3.543)	
≥5	32	12 (37.5)	1.332 (0.530–3.346)		6 (18.8)	1.808 (0.526–6.213)		6 (18.8)	1.825 (0.531–6.272)	
Cessation groups ^g^										
Group A	106	36 (34.0)	1	1	16 (15.1)	1	1	15 (14.2)	1	1
Group B	33	12 (36.4)	1.117 (0.494–2.53)	1.054 (0.421–2.643)	3 (9.1)	0.576 (0.157–2.123)	0.817 (0.206–3.243)	3 (9.1)	0.619 (0.167–2.293)	0.934 (0.226–3.852)

^a.^ Model 1: Adjusted for age. ^b.^ Model 2: Adjusted for age, nicotine dependence, alcohol consumption, number of triggers for smoking, self-efficacy, and cessation group. ^c.^ Nicotine dependence: mild (0–3), moderate (4–6), and severe (7–10), using Fagerström test for nicotine dependence. ^d.^ Triggers for smoking: drinking coffee or alcohol, seeing others smoking in real life or on television, being stressed, excited or tired, being alone, and other daily activities such as after walking, before going to bed, after taking a shower, during phone calls, and while playing a game. ^e.^ Self-efficacy: using Self-efficacy Scale. ^f.^ Reasons to quit: identifying themselves as smokers, having self-confidence for quitting, personal health issue related to tobacco use, economic burden from buying tobacco, having a good social relationship, getting positive public attention, recommendation to quit from their surroundings, and others. ^g.^ Cessation groups: Group A offers 1-year counseling for quit and maintenance; Group B offers 4 weeks counseling for motivational interviewing and then 1-year counseling for quit and maintenance.
